# Educational Case: Mesothelioma

**DOI:** 10.1016/j.acpath.2025.100239

**Published:** 2026-02-10

**Authors:** Kassaye Firde, Deborah J. Chute

**Affiliations:** a Resident Physician, Temple University Hospital, Department of Pathology, Philadelphia, PA, USA; b Professor of Pathology, Cleveland Clinic Lerner College of Medicine, Cleveland Clinic Department of Pathology, Cleveland, OH, USA

**Keywords:** Pathology competencies, Organ system pathology, Respiratory system, Pleural disorders, Pleural effusion, Pleural neoplasms, Mesothelioma, Cytology, Ancillary studies

The following fictional case is intended as a learning tool within the Pathology Competencies for Medical Education (PCME), a set of national standards for teaching pathology. These are divided into three basic competencies: Disease Mechanisms and Processes, Organ System Pathology, and Diagnostic Medicine and Therapeutic Pathology. For additional information, and a full list of learning objectives for all three competencies, see https://doi.org/10.1016/j.acpath.2023.100086

## Primary objective

Objective RS7.2: Pleural Neoplasms. Describe the clinicopathologic findings of neoplasms involving the pleura and pleural cavity.

Competency 2: Organ System Pathology; Topic: RS: Respiratory System; Learning Goal 7: Pleural Disorders.

## Secondary objective

Objective CYP2.2: Ancillary Testing. Describe how ancillary testing such as flow cytometry, immunohistochemistry, and molecular diagnostic testing are used in conjunction with cytology examination.

Competency 3: Diagnostic Medicine and Therapeutic Pathology; Topic: CYP: Cytopathology; Learning Goal 2: Cytologic Diagnosis.

## Patient presentation, Part 1

A 75-year-old man with a past medical history of high blood pressure and type II diabetes mellitus presents to the emergency department with a one-month history of worsening nonproductive cough, dull right-sided chest wall pain and shortness of breath. He has a 10-pack year smoking history and quit 20 years ago. He is currently retired but previously worked in the construction industry.

## Diagnostic findings, Part 1

On physical examination, he is in mild respiratory distress; vital signs show blood pressure of 140/90 mmHg, respiratory rate 32 breaths/minute, pulse rate of 110 beats/minute, temperature 98.5 ^O^F and oxygen saturation of 89 %. Cardiac examination shows normal S1 and S2, regular rate and rhythm without murmurs, heaves or thrills. Chest examination reveals decreased breath sounds over the right lung base with dullness to percussion and reduced tactile fremitus. Left chest examination is normal. His abdomen is soft and non-tender with bowel sounds present.

## Questions/discussion points, Part 1

### What differential diagnosis does his physical exam and history suggest?

The physical examination demonstrates respiratory distress (elevated respiratory rate, elevated blood pressure and pulse and reduced oxygenation) along with reduced breath sounds in the right lung base. Reduced breath sounds can be caused by reduced sound generation (such as not breathing deeply or obstructive airway disease leading to reduced air movement) or reduced sound transmission (such as a pleural effusion or pneumothorax). In this case, the dullness to percussion is suggestive of a pleural effusion (as a pneumothorax typically causes hyperresonance). The most common causes of pleural effusion include fluid overload states (such as congestive heart failure, nephrotic syndrome, or chronic liver disease) or inflammatory states (such as pneumonia, systemic connective tissue diseases, chest trauma and malignancies). Most fluid overload states cause bilateral effusions. When a pleural effusion is unilateral and large, localized conditions like tumors, trauma and pneumonia are more likely; given the patient's history of smoking and potential work exposures, malignancy is a significant concern.

## Diagnostic findings, Part 2

The patient's complete blood count with differential, basic metabolic profile, and arterial blood gas analysis are provided in Table 1, which are within the normal range. Chest X-ray reveals a unilateral moderate to large right pleural effusion.

**Table 1:** Initial peripheral blood laboratory test results.

Laboratory Test	Patient Value	Reference range
**Complete blood count**		
RBC count (cells/mm^3^)	4.7	3.5–5.5
Hemoglobin (g/dL)	14.5	13.5–17.5
Hematocrit (%)	44	41–53
WBC count (cells/mm^3^)	6500	4500–11,000
Platelet count (cells/mm^3^)	250000	150,000–400,000
**WBC differential**		
Segmented neutrophils (%)	56	54–62
Band neutrophils (%)	4	3–5
Lymphocytes (%)	32	25–33
Monocytes (%)	6	3–7
Eosinophils (%)	1	1–3
Basophils (%)	0.4	0–0.75
**Arterial blood gas**		
pH	7.38	7.35–7.45
PaCO_2_ (mmHg)	42	33–45
PaO_2_ (mmHg)	76	75–105
Bicarbonate (mmol/L)	24	22–28
**Basic metabolic panel**		
Glucose (mg/dL)	98	70–100
Blood urea nitrogen (mg/dL)	9	7–18 mg/dL
Creatinine (mg/dL)	1.1	0.6–1.2
eGFR	69	>60 mL/min/1.73m^2^
Sodium (mmol/L)	140	136–145
Potassium (mmol/L)	3.8	3.5–5.0
Chloride (mmol/L)	104	95–105
Calcium (mg/dL)	8.6	8.4–10.2
Total protein (g/dL)	6.1	6.0–7.8
Albumin (g/dL)	4.0	3.5–5.5
LDH (U/L)	150	130–270

Abbreviations: RBC: red blood cell; WBC: White Blood cell; eGFR: estimated Glomerular filtration Rate; LDH: Lactate dehydrogenase.

## Questions/discussion points, Part 2

### What are the best additional tests to perform in the workup of a unilateral pleural effusion?

With initial imaging showing a unilateral effusion on chest X-ray, the next most common imaging to perform is a chest CT scan; this can identify additional features of the ongoing process including pulmonary edema, cardiac abnormalities, pneumonia, blood clots or mass lesions. Thoracentesis is both a diagnostic and therapeutic tool; it allows both pleural fluid biochemical analysis and cytologic examination for malignancy, and additionally provides symptomatic relief in patients with significant pleural effusions. Biochemical analysis of pleural fluid helps identify effusions as transudates or exudates, which narrows the differential diagnosis. Cytological evaluation of pleural fluid remains the primary diagnostic tool in suspected malignancy, as exfoliated cells provide a representative sample from the entire pleural cavity. While thoracocentesis with pleural cytology is minimally invasive and cost-efficient, the disadvantage includes lower diagnostic accuracy for certain malignancies such as sarcomatoid mesotheliomas and hematolymphoid malignancies. Ultrasound and CT-guided pleural biopsies are more invasive with procedure-related risks, but can be more definitive if malignancy is suspected. Thoracoscopic guided biopsy is the gold standard as it allows the operator to explore the contents of the pleural cavity, guide biopsy to the most grossly pathologic areas under direct vision, remove pleural fluid and perform pleurodesis.^1^^,^^2^

## Diagnostic findings, Part 3

**Fig. 1:**
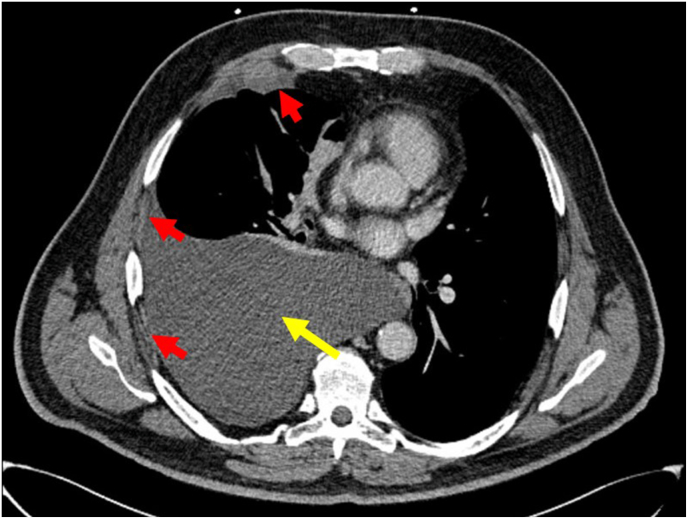
**Chest Computed Tomography (CT)**: Large right pleural effusion (yellow arrow), with numerous scattered right-sided nodular pleural thickening, as well as a dominant pleural nodule inferiorly measuring approximately 4.3 × 3.5 cm (red arrows).

Chest Computed tomography (CT) scan (Fig. 1) shows a large right pleural effusion, with numerous scattered small right-sided nodular areas of pleural thickening as well as a dominant pleural nodule inferiorly, measuring approximately 4.3 × 3.5 cm. Ultrasound-guided thoracocentesis is performed under sterile conditions. The findings of the pleural fluid analysis are summarized in Table 2. The findings of pleural fluid analysis are interpreted according to the Light's criteria as outlined below in Table 3.

**Table 2:** Pleural fluid analysis results.

Fluid color	Turbid
Fluid volume	100 ml
RBC count (cells/mm^3^)	2600
Total WBC count (cells/mm^3^)	810/mm^3^
Neutrophils (%)	5
Lymphocytes (%)	22
Histiocytes (%)	73
Glucose (mg/dL)	93
Protein (g/dL)	4.6
LDH (Unit/L)	752

**Table 3:** Light's criteria for an exudative effusion, with patient results and calculation of ratios.

Light's criteria for exudative effusions (must meet at least one)	Patient results	Patient interpretation
Pleural fluid protein/serum protein ratio greater than 0.5	Pleural fluid protein: 4.6 g/dL	Ratio: 0.72
	Serum protein: 6.4 g/dL	
Pleural fluid LDH/serum LDH ratio greater than 0.6	Pleural fluid LDH: 752 U/L	Ratio: 5.0
	Serum LDH: 150 U/L = 5.0	
Pleural fluid LDH level is greater than two-thirds (67 %) of the normal upper limit for serum LDH	Pleural fluid LDH: 752 U/L	Abnormal, as above 180 U/L
	Serum LDH normal upper limit: 270 U/L	

## Questions/discussion points, Part 3

### Exudate versus transudate: why does it matter?

Pleural effusions are classified clinically as transudative or exudative. A transudate is a type of fluid resulting from an imbalance of hydrostatic and oncotic pressures in patients with congestive heart failure, cirrhosis, hypoalbuminemia, and nephrotic syndrome, resulting in migration of water and electrolytes from tissue into a third space. In contrast, exudates are the result of injury as occurs with infection, malignancy, immunologic responses or trauma, including pulmonary infarction and lymphatic abnormalities. The injury allows fluid that includes water, electrolytes, protein, and cellular components to migrate across compartments. Laboratory evaluation starts with gross examination as color and character of fluid give important clues. The next step of routine pleural fluid analysis tests includes cell counts and cell differential, total protein, lactate dehydrogenase (LDH) and glucose. The distinction between exudate and transudate is important because pleural involvement by malignancy or inflammatory diseases causes an exudate that often requires cytological examination. Light's criteria (Table 3) are a simple and accurate method used to help classify effusions as transudate or exudate, using readily accessible pleural fluid and serum parameters. If at least one of Light's criteria is present, the effusion is classified as an exudate. Limitations of the criteria include lower specificity, the need for blood sampling and duplicative use of highly correlated criteria (e.g., pleural fluid LDH and pleural fluid-to-serum LDH). Despite the limitations of Light's criteria (lower specificity), it remains widely used.^3^

Additional laboratory investigations for exudates can be tailored to the underlying clinical suspicion as summarized in Table 4.

**Table 4:** Differential diagnosis for exudates and specific laboratory tests to consider.

Exudate Differential	Laboratory tests to consider
Infection	pH, gram stain, acid-fast Bacilli (AFB) and culture
Malignancy	Cytology, flow cytometry (for suspected lymphoma or multiple myeloma)
Connective tissue disease	Cytology, rheumatoid factor, antinuclear antibody testing
Iatrogenic (esophageal perforation, hemothorax, chylothorax …)	pH, hematocrit, amylase, cholesterol and triglyceride levels

### What is the role of cytology in the evaluation of pleural effusions?

Cytologic examination of pleural fluid involves concentrating the cellular component of the effusion fluid and making a preparation for review under a microscope. This can be done in several ways, including cytospin with a modified Wright-Giemsa stain, or Papanicolaou stain of a smear or liquid-based preparation such as ThinPrep. Direct examination of the cellular component allows assessment for the cells to assess for malignancy. Cytology is the most sensitive test in the early detection of pleural malignancy, particularly in the assessment of metastatic disease to the pleura. Cytology is more sensitive than blind biopsy for detecting serosal malignancy (71 % vs 45 %), presumably because the fluid sample provides a more representative sample.^4^ The overall diagnostic sensitivity and specificity of pleural fluid cytology for malignant pleural effusion are 58.2 % and 97.0 %, respectively. The sensitivity is particularly high for lung adenocarcinoma (83.6 %), ovarian cancer (85.2 %) and breast cancer (65.3 %). In contrast, sensitivity is lower for mesothelioma (28.9 %) and lung squamous cell carcinoma (24.2 %).^5^

When collecting pleural fluid for a suspected diagnosis of malignancy, it is recommended to send as much fluid volume as reasonably possible for cytologic evaluation and ancillary tests.^6^ Studies have shown that a fluid volume of at least 75 ml is required to decrease the risk of a false-negative result.^7^ In situations with a high suspicion for malignancy and negative cytological results, multiple sequential high-volume specimens should be examined. Repeat cytologic analysis may identify tumor cells in additional specimens due to intermittent shedding of tumor cells in the pleural effusion.

### What are the most common neoplasms that involve the pleura?

Secondary involvement of the pleura by other neoplasms is by far the most common cause of malignancy in the pleura, and can include direct extension by a lung carcinoma, metastatic carcinomas (most commonly breast or ovarian, but can be from any site/primary), or involvement by systemic lymphomas.^8^ Typically, these present as unilateral or bilateral effusions in a patient with a clinical history of prior malignancy. Imaging may show multiple pulmonary nodules (in the case of metastasis) or a solitary lung nodule (in the case of a primary lung neoplasm or oligometastatic disease) with or without lymphadenopathy or may lack other findings.

Mesothelioma is the most common primary tumor of the pleura and typically presents as an unexplained unilateral effusion, accompanied by a pleural-based mass, pleural studding, or diffuse pleural thickening.

Rarely, other primary neoplasms occur in the pleura. Solitary fibrous tumor is the most common benign neoplasm of the pleura, and presents classically as a pleural-based smooth-bordered mass. Other mesenchymal neoplasms are very rarely seen (such as calcifying fibrous tumor, desmoid-type fibromatosis, epithelioid hemangioendothelioma, angiosarcoma, synovial sarcoma, desmoplastic round cell sarcoma, and pleuropulmonary blastoma). These mesenchymal neoplasms almost never shed into pleural effusions. Primary lymphomas of the pleural space are typically either an HHV-8 related primary effusion lymphoma or a diffuse large B-cell lymphoma; these are commonly identified initially on effusion cytology. Primary effusion lymphoma occurs in immunosuppressed patients, and initially was associated with HIV, but is also seen in elderly patients with HHV8 in endemic areas; it typically presents as unexplained unilateral or bilateral effusions. Diffuse large B-cell lymphoma (usually associated with chronic inflammation) typically develops in patients with a long-standing history of chronic empyema/pyothorax, and is associated with EBV; this typically presents with a pleural-based mass and invasion of nearby structures.^9^

## Diagnostic findings, Part 4

**Fig. 2:**
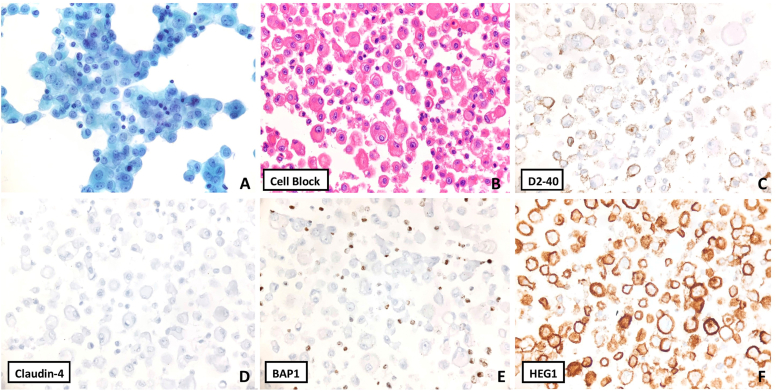
**Mesothelioma. A**, ThinPrep (Papanicolaou stain, 400x) shows a highly cellular sample, with relatively bland cells showing mesothelial-type features with mildly atypical features (hypercellularity, enlarged nucleoli and occasional vacuolization). B. Cell Block (H&E, 400x) also shows a cell-in-cell pattern which is concerning for malignancy. **C–F,** IHC stains were performed with adequate controls on the cell block material (400x). D2-40 shows patchy membranous staining. Claudin 4 was negative. BAP1 shows loss in the nuclei of the mesothelioma cells, but retained staining in the background inflammatory cells. HEG1 shows strong diffuse membranous staining. The combined cytologic features and IHC profile were diagnostic of mesothelioma.

Given the presence of an exudative pleural effusion and imaging showing pleural nodularity, a cytologic examination is performed. ThinPrep, Cytospin and cell block materials were prepared. The pleural effusion cytology (Fig. 2) shows a highly cellular sample, with relatively bland cells showing mesothelial-type features (large loose clusters with mulberry borders along with many single cells in the background, lacy skirt, central to slightly eccentric nuclei, dense moderate central to eccentric cytoplasm, smooth nuclear borders, fine chromatin) along with mildly atypical features (enlarged nucleoli, highly cellular sample, and occasional vacuolization). Immunohistochemical (IHC) stains were performed with adequate controls on the cell block material. BAP1 shows loss in the nuclei of the mesothelioma cells, but retained staining in the background inflammatory cells. HEG1 shows strong diffuse membranous staining. D2-40 shows patchy membranous staining. Claudin 4, TTF-1, Napsin A, p40, MOC31 were negative. The combined cytologic features and IHC profile are diagnostic of mesothelioma.

## Questions/discussion points, Part 4

### What normal cells are found in a pleural effusion?

**Fig. 3:**
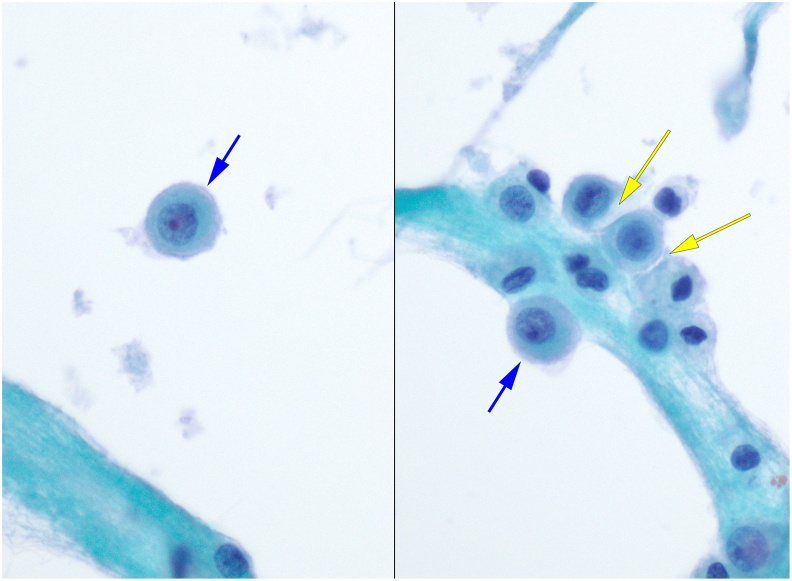
Normal mesothelial cells in pleural fluid show central nuclei with small nucleoli, and central dense cytoplasm with a peripheral “lacy skirt” (blue arrows) caused by the surface microvilli, and when found in clusters the microvilli push cells apart, with a resulting “window” between the cells (yellow arrows) (Papanicolaou stain, 600x, ThinPrep).

Normal cells found in pleural effusions include mesothelial cells, histocytes and lymphocytes. Normal mesothelial cells are round with a central nucleus and have abundant cytoplasm, arranged as single cells or in small clusters (Fig. 3). Characteristically, mesothelial cells have long slender microvilli on their surface (not visible on regular microscopy, but seen on electron microscopy), giving their cytoplasm a pale outer rim classically called a “lacy skirt.” Additionally, the microvilli prevent cells from aggregating closely, with a resulting space, or “window” between two adjacent cells. When present in clusters, mesothelial cells typically have a “knobby” or “mulberry” border due to the lack of tight junctions and presence of microvilli. Histocytes typically show foamy cytoplasm with a round to indented nucleus either centrally or eccentrically located.

### What are reactive mesothelial cells?

In the setting of an acute or chronic injury, mesothelial cells can show a wide range of reactive morphological appearances. These include nuclear pleomorphism, binucleation or multinucleation, reduced cytoplasm resulting in an increased nuclear-to-cytoplasmic ratio, coarse chromatin, irregular nuclear contours, prominent nucleoli and cytoplasmic vacuolization.^10^ Not surprisingly, these morphological features often overlap with features of malignancy (eg, metastatic adenocarcinoma, mesothelioma), creating diagnostic difficulty.

### What are the classic features of metastatic adenocarcinoma in an effusion?

**Fig. 4:**
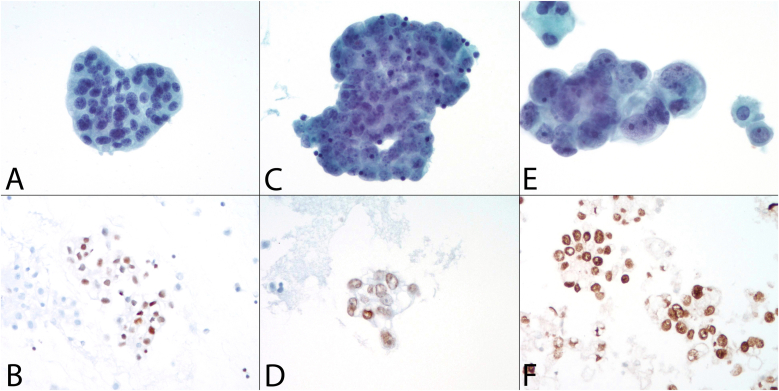
**Metastatic Adenocarcinoma in Pleural Effusion**. **A.** Metastatic breast carcinoma in pleural fluid exhibiting characteristic “cannonball” appearance (ThinPrep, Papanicolaou stain, 400x). **B.** GATA-3 nuclear positivity in the cell block supports breast origin (400x). **C.** Metastatic high-grade serous carcinoma with psammomatous calcification (ThinPrep, Papanicolaou stain, 400x). **D.** PAX-8 nuclear positivity in the cell block supports Mullerian origin (400x). **E.** Metastatic lung adenocarcinoma with adjacent mesothelial cells (ThinPrep, Papanicolaou stain, 400x). **F.** Nuclear TTF-1 positivity supports a diagnosis of lung adenocarcinoma (400x).

Classically, metastatic adenocarcinoma (Fig. 4) is identified by finding a second population of cells, different from background normal mesothelial cells, in the effusion. Often, metastatic adenocarcinomas will create three-dimensional clusters with a smooth so-called “community” border or edge. The morphologic characteristics will vary depending on the specific type of metastatic adenocarcinoma, but common features include increased nuclear-to-cytoplasmic ratio, nuclear pleomorphism, coarse chromatin and prominent nucleoli. The presence of intracytoplasmic vacuoles that compress the nucleus should prompt consideration for adenocarcinoma. However, well-known exceptions to these features exist; the most common exceptions are lobular breast cancer and signet ring cells of gastric cancer origin, which typically present as single cells with less atypia and can blend with normal mesothelial cells, making the diagnosis challenging.

### What are the classic features of mesothelioma?

**Fig. 5:**
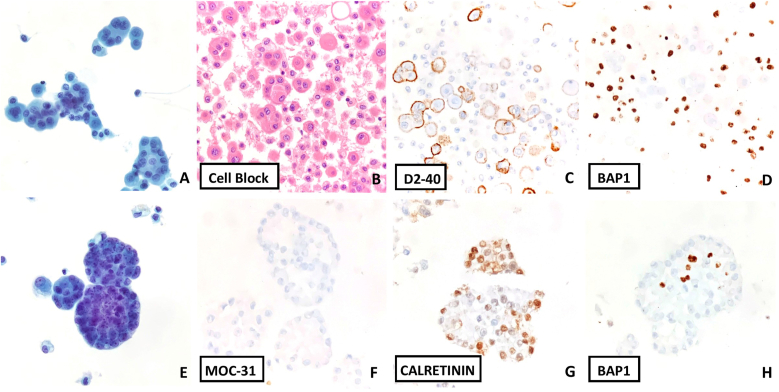
**Patterns of Mesothelioma on Cytology**. **A-D**, showing cells with bland morphology (looking like normal mesothelial cells – lower cellularity, smaller clusters and single cells, “mulberry” borders to clusters, lacy skirt edges, round smooth nuclei and pinpoint to no nucleoli). However, BAP1 loss (with retained staining in adjacent normal inflammatory cells) and mesothelial staining pattern for IHC (positive for D2-40) – was a feature of an epithelioid mesothelioma. A concurrent pleural biopsy confirmed the diagnosis (A ThinPrep, Papanicolaou stain, 400x, B. Cell block, H&E, 400x, C, D. Cell block, 400x). **E-H**, Challenging case that looked like adenocarcinoma rather than mesothelioma, given the community borders forming “cannonball”-like structures. However, the IHC proved mesothelial origin (positive for calretinin, negative for MOC31) and BAP1 loss (E. ThinPrep, 400x, Papanicolaou stain, 400x, F–H cell block, 400x).

Mesothelioma is a heterogeneous category of tumors, which includes epithelioid, sarcomatoid, and mixed patterns. Generally, only epithelioid mesotheliomas will shed cells into the effusion fluid, so that most mesotheliomas on cytology have an epithelioid morphology. The most classic features of mesothelioma on effusion cytology are atypical cells that look similar to mesothelial cells (lacking community borders, with windows and centrally located nuclei), but with a highly cellular sample and containing cells arranged in very large clusters (>50 cells) (so called “mulberry” pattern) (Fig. 5). However, mesotheliomas can become sufficiently atypical that they lose the typical morphology of mesothelial cells; in this situation recognition of involvement by malignancy is quickly done, but identification of cell type and origin will require ancillary testing as discussed below.

### What ancillary studies are used in pleural fluid cytology?

The most commonly used ancillary studies in the evaluation of pleural fluid cytology are immunohistochemical (IHC) stains. These stains can be used in different ways, depending on the specific differential diagnosis considered. The two most common differentials are 1) differentiating adenocarcinoma from mesothelioma, and 2) differentiating reactive mesothelial cells from mesothelioma.

### Differentiating adenocarcinoma from mesothelioma

Even if specific morphologic features of mesothelioma or metastatic adenocarcinoma are seen in pleural effusion cytology, it is recommended to do confirmatory IHC. It is recommended to do at least two mesothelial markers and two carcinoma markers to minimize the risk of a false negative result.^11^ The best markers for mesothelial differentiation include calretinin, WT1 (Wilms tumor 1), D2-40 (Podoplanin), and HEG1. However, it is important to note that these markers are not entirely specific; focal staining for any of these markers can be seen in various adenocarcinomas.

Calretinin has a sensitivity and specificity of 91 % and 96 %, respectively, in differentiating mesothelioma from metastatic carcinoma in serous effusions.^12^ WT-1 has a sensitivity and specificity of 100 % in distinguishing mesothelioma from lung squamous cell carcinoma and adenocarcinoma.^13^ However, studies have shown that WT-1 can be positive in other metastatic adenocarcinomas, most of which are Müllerian origin.^14^ D2-40 is expressed in 94 % of mesotheliomas. Notably, D2-40 also stains 7 % of ovarian adenocarcinoma.^15^ HEG1 is a newly developed marker for epithelioid mesothelioma; to date, it has been reported to have a sensitivity of 94 % and specificity of 88 % for epithelioid mesothelioma, but also is expressed in 44 % of sarcomatoid mesotheliomas and 76.9 % of reactive mesothelial cells. Notably, a small subset of ovarian carcinomas can also show HEG1 staining.^16^

While tumor-specific immunohistochemical stains exist for various adenocarcinomas (i.e. TTF-1 for lung adenocarcinoma), it is helpful to have IHC stains that will frequently stain most adenocarcinomas regardless of site of origin, but will be negative in benign mesothelial cells and mesothelioma. These screening general adenocarcinoma markers are critical to prevent missing a metastasis. Many markers have been developed over the years, and include Claudin-4, BerEP4, MOC31, B72.3 and LEUM1.

Claudin-4 IHC has been reported to distinguish adenocarcinoma from mesothelioma with a sensitivity of 98 % and specificity of 99 %.^17^ The sensitivity of MOC-31 for metastatic adenocarcinoma was 99 % and the specificity was 77 %.^18^ The sensitivity and specificity of Ber-EP4 in the diagnosis of metastatic adenocarcinoma was 97 % and 100 %, respectively.^19^ LEUM1 is an older marker that has fallen out of general use, but shows 77 % sensitivity for adenocarcinoma, and 93 % specificity.^20^

Which markers are used in any given case is typically related to the availability of cytology-validated IHC stains in a given laboratory, but a commonly used example panel would be calretinin, D2-40, Claudin-4 and MOC31 to screen for adenocarcinoma in a pleural effusion. Once confirmation of metastatic adenocarcinoma is made, additional site-specific IHCs can be used to narrow down or confirm a suspected primary site**.**

### Distinguishing mesothelioma from reactive mesothelial cells

The distinction between mesothelioma and reactive mesothelial cells is morphologically very challenging, but ancillary studies can be helpful. BRCA-associated protein 1 (BAP1) is altered in 60–79 % of mesotheliomas, resulting in a loss of nuclear expression. In contrast, BAP1 shows retained nuclear expression in benign mesothelial cells in nearly all cases. Therefore, loss of BAP1 expression is strong evidence for a mesothelioma.^21^ However, note should be made that BAP1 loss can be seen in other tumors, so it is not useful in the differential diagnosis between adenocarcinoma and mesothelioma. Another important gene is the CDKN2A locus on chromosome 9p.21, which encodes the p16 protein tumor suppressor gene. Up to 80 % of pleural mesothelioma have homozygous deletion in the CDKN2A gene. Thus, fluorescence in situ hybridization (FISH) studies can be performed in those challenging cases with retained BAP1, and the loss of both copies of this gene supports a diagnosis of mesothelioma. Unfortunately, IHC for p16 loss is not specific for CDKN2A homozygous deletion, and FISH is the recommended modality for this testing.^22^

Older stains like desmin and epithelial membrane antigen (EMA) were previously used to distinguish reactive mesothelial cells from mesothelioma but have been largely supplanted by the newer markers discussed above. Reactive mesothelial cells show staining with desmin, while EMA stains malignant mesothelial cells. In summary, an IHC panel including BAP1 with addition of FISH for CDKN2A loss, or older markers including desmin and EMA, can be used to distinguish mesothelioma from reactive mesothelial cells.

## Diagnostic findings, Part 5

The patient subsequently underwent right thoracotomy, total radical pleurectomy and decortication.

### Describe the histologic features of the resected specimen?

**Fig. 6:**
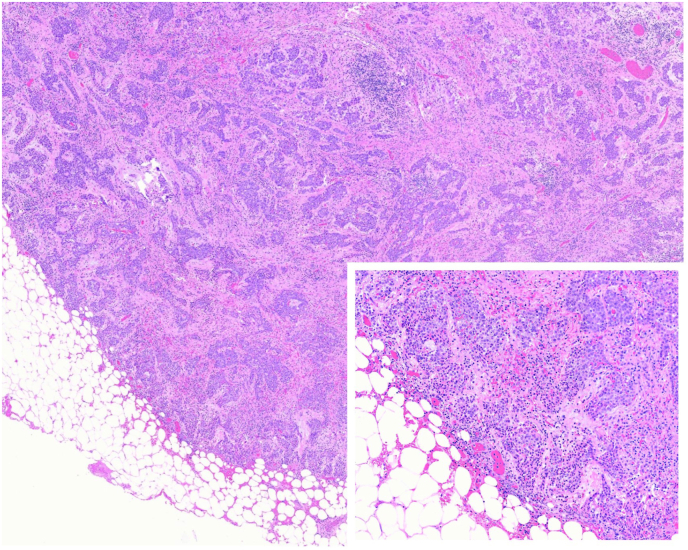
**Epithelioid Mesothelioma (H&E, 40x).** Malignant cells with trabecular growth patterns infiltrating into adjacent adipose tissue. H&E, 200x (inset) shows malignant cells with eosinophilic cytoplasm, round nuclei with vesicular chromatin and small nucleoli.

Microscopic examination of the resection specimen shows infiltrative epithelioid malignant cells with trabecular and solid architecture involving the underlying lung tissue and chest wall (Fig. 6). The tumor cells show severe nuclear atypia including high pleomorphism, frequent mitosis and scattered foci of necrosis.

### What is the diagnosis?

The histologic features are diagnostic of a high-grade, diffuse mesothelioma, epithelioid type. The pathologic stage was pT3N0M0. The patient was subsequently treated with several cycles of adjuvant chemotherapy and radiotherapy.

## Questions/discussion points, Part 5

### What are the risk factors for mesothelioma?

Most mesotheliomas are linked to asbestos exposure, particularly from the commercial forms like amosite and crocidolite, with occupational exposure accounting for 80–90 % of cases in men compared to 20–40 % in women.^23^ The most common occupations associated with asbestos exposure include asbestos mining, construction work (building demolition, insulation, flooring and roofing) and shipyard workers. Erionite, a non-commercial mineral fiber found in soil and rocks, causes a high incidence of mesothelioma in the Cappadocia region of Turkey, central Mexico and North Dakota in the US. The disease typically develops 20–40 years postexposure. The mechanism of asbestos causing mesothelioma involves mineral fibers inducing chronic inflammation and DNA damage, which is potentiated in genetically susceptible individuals like those with BAP1 tumor predisposition syndrome.^24^

### What are the histological subtypes of mesothelioma?

Mesotheliomas can be localized or diffuse. Diffuse mesothelioma shows widespread involvement of the pleura or other serosal surfaces, while localized mesothelioma radiologically shows a circumscribed tumor often mistaken for primary sarcomas or carcinoma. Histologically, mesotheliomas are classified as epithelioid, sarcomatoid (including desmoplastic), and biphasic subtypes.

Epithelioid mesotheliomas are the most common subtype, showing tubulopapillary, trabecular, micropapillary, and solid architectural patterns. The cells exhibit abundant eosinophilic cytoplasm, with round nuclei, vesicular chromatin and small nucleoli. The cells may have a histiocytoid appearance and background fibrous stroma can vary from scant to prominent. These tumors are graded as low- or high-grade, depending on nuclear atypia, mitotic count and presence of necrosis. Epithelioid mesotheliomas are more likely to shed in pleural effusions and show overlap cytologically and immunohistochemically with metastatic carcinomas.

**Fig. 7:**
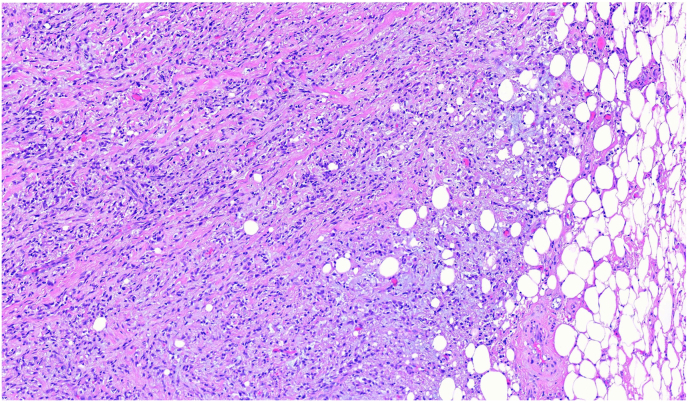
**Sarcomatoid Mesothelioma**. Malignant spindle cells arranged in haphazard patterns with fibro-myxoid stroma invading adjacent adipose tissue (H&E, 200x).

Sarcomatoid mesotheliomas (Fig. 7) are the second most common subtype, showing fascicles or haphazard patterns of spindle cells invading surrounding adipose tissue or lung parenchyma. These tumors have atypical mitotic figures and necrosis, and in rare cases exhibit heterologous components like osteosarcomas, rhabdomyosarcoma or chondrosarcoma. Sarcomatoid mesotheliomas almost never shed in pleural fluid making their diagnosis difficult in effusion specimens which typically contain only reactive mesothelial cells. The main differential diagnosis for sarcomatoid mesothelioma includes metastatic sarcomatoid carcinomas from lung, kidney and other sites. Biphasic mesotheliomas are defined as a mesothelioma with at least 10 % epithelioid and 10 % sarcomatoid components each in a resection specimen.

The overall survival in patients with mesothelioma is poor. Young age, epithelioid histology and early stage are indicators of longer survival periods.^25^

## Teaching points

• Pleural effusions are classified clinically as transudative or exudative. Transudates result from an imbalance of hydrostatic and oncotic pressures, while exudates result from injury to the mesothelium.
• An exudative effusion is defined as one with an elevated protein and LDH level compared to the serum levels.
• As a minimally invasive and cost-efficient procedure, cytological evaluation of pleural fluid remains the primary diagnostic tool in suspected malignancy.
• In a suspected diagnosis of malignancy, it is recommended to send at least 75 ml of effusion sample to decrease the risk of false-negative results.
• Normal cytological features of pleural effusions include mesothelial cells, histocytes and lymphocytes.
• Normal mesothelial cells are round with a central nucleus and abundant cytoplasm. Characteristically, mesothelial cells have a peripheral “lacy skirt” and spaces between cells, or “windows.”
• The classic finding of metastatic adenocarcinoma in an effusion is a “second’’ or “foreign” population of cells, often with “community” borders.
• Classically, on cytology mesothelioma shows a highly cellular sample showing large clusters (>50 cells) of atypical cells with windows and centrally located nuclei (so called “mulberry” pattern).
• A panel of immunohistochemical stains that includes at least 2 mesothelial markers (calretinin, HEG1, WT1, D2-40, etc.) and 2 general adenocarcinoma markers (claudin-4, MOC31, BerEP4, etc.) is needed to distinguish adenocarcinoma from benign mesothelial cells and mesothelioma.
• To distinguish mesothelioma from reactive mesothelial cells, an IHC panel including BAP1, and addition of FISH for CDKN2A loss, can be used to help make the diagnosis.
• Most mesotheliomas are linked to asbestos exposure and the disease typically develops 20–40 years postexposure.
• Mesotheliomas can be localized or diffuse, based on radiologic features. Histologically, mesotheliomas are classified as epithelioid, sarcomatoid (including desmoplastic), and biphasic subtypes.

## Funding

The article processing fee for this article was funded by an Open Access Award given by the Society of ‘67, which supports the mission of the Association for Academic Pathology to produce the next generation of outstanding investigators and educational scholars in the field of pathology. This award helps to promote the publication of high-quality original scholarship in *Academic Pathology* by authors at an early stage of academic development.

## Declaration of conflicting interests

The authors declare that they have no known competing financial interests or personal relationships that could have appeared to influence the work reported in this paper.
